# Case series: successful salvage from refractory cardiogenic shock caused by COVID-19-associated myocardial injury with temporary ventricular assist devices

**DOI:** 10.1093/ehjcr/ytae308

**Published:** 2024-06-29

**Authors:** Kohei Tonai, Keiko Ohta-Ogo, Satoshi Kainuma, Naoki Tadokoro, Yasumasa Tsukamoto, Kinta Hatakeyama, Tadaki Suzuki, Satsuki Fukushima

**Affiliations:** Department of Cardiovascular Surgery, National Cerebral and Cardiovascular Center, 6-1 Kishibe-Shinmachi, Suita, Osaka 564-8565, Japan; Department of Pathology, National Cerebral and Cardiovascular Center, 6-1 Kishibe-Shinmachi, Suita, Osaka, Japan; Department of Cardiovascular Surgery, National Cerebral and Cardiovascular Center, 6-1 Kishibe-Shinmachi, Suita, Osaka 564-8565, Japan; Department of Cardiovascular Surgery, National Cerebral and Cardiovascular Center, 6-1 Kishibe-Shinmachi, Suita, Osaka 564-8565, Japan; Department of Transplant Medicine, National Cerebral and Cardiovascular Center, 6-1 Kishibe-Shinmachi, Suita, Osaka, Japan; Department of Pathology, National Cerebral and Cardiovascular Center, 6-1 Kishibe-Shinmachi, Suita, Osaka, Japan; Department of Pathology, National Institute of Infectious Diseases, Toyama 1-23-1, Shinjuku-ku, Tokyo, Japan; Department of Cardiovascular Surgery, National Cerebral and Cardiovascular Center, 6-1 Kishibe-Shinmachi, Suita, Osaka 564-8565, Japan

**Keywords:** COVID-19, Mechanical circulatory support, Ventricular assist device, Cardiogenic shock, Biventricular failure, Case series

## Abstract

**Background:**

Coronavirus disease 2019 (COVID-19) is predominantly known to cause respiratory injury; however, the present case series highlights four instances in which the infection resulted in significant cardiac complications. Among such cases, some represent severe cardiogenic shock, which necessitates the immediate introduction of mechanical circulatory support (MCS) for salvage.

**Case summary:**

This case series involved patients with COVID-19-associated myocardial injury leading to fulminant cardiogenic shock. These patients required immediate implementation of peripheral MCS, followed by an instant upgrade to a central MCS system due to anatomical limitations and severe biventricular dysfunction. Central MCS provided effective ventricular unloading, resulting in a significant and prompt improvement in ventricular function. The treatment timeline showed rapid deterioration followed by remarkable recovery within 2 weeks of MCS initiation, demonstrating the effectiveness of aggressive and tailored MCS strategies in managing severe COVID-19-related cardiac complications.

**Discussion:**

This study provides significant insights into the cardiovascular implications of COVID-19, particularly in the context of severe myocardial injury that leads to cardiogenic shock. The report underscores the importance of early recognition and intervention in such cases, focusing on the use of MCS as a life-saving modality. The findings also revealed unique pathological features of COVID-19-associated myocardial injury, including macrophage-predominant infiltration and microthrombosis, which are distinct from the features of conventional myocarditis. These findings highlight the need for further research on the pathophysiology of COVID-19-related cardiac injuries and the development of targeted therapeutic strategies.

**Learning points**

- Rapid haemodynamic deterioration in coronavirus disease 2019 (COVID-19)-associated refractory cardiogenic shock underscores the need for urgent initiation of mechanical circulatory support.
- Central mechanical circulatory support induction yielded significant improvements in ventricular function within 2 weeks, suggesting the importance of ventricular unloading in managing COVID-19-related cardiogenic shock.
- Microthrombosis with endothelial activation, together with macrophage-dominant infiltration, constitutes the principal pathological feature characterizing COVID-19-related myocardial injury.

## Introduction

Coronavirus disease 2019 (COVID-19), caused by severe acute respiratory syndrome coronavirus 2 (SARS-CoV-2), affects the respiratory and cardiovascular systems, resulting in arrhythmias, myocarditis, and heart failure. In this study, we report four cases of fulminant cardiogenic shock resulting from COVID-19-associated myocardial injury. All patients required immediate implantation of peripheral mechanical circulatory support (MCS) and a subsequent system upgrade to central support owing to anatomical restrictions.

Contrary to expectations, within 2 weeks, ventricular function had improved dramatically, enabling prompt removal of the devices. Myocardial histopathological features, including macrophage-predominant infiltration and the formation of microthrombi without significant necrotic changes in the myocardium, constituted the key features of COVID-19-associated myocyte injury, which is distinct from conventional fulminant myocarditis and implies reversibility of ventricular function.^1^

## Summary figure

**Figure:**
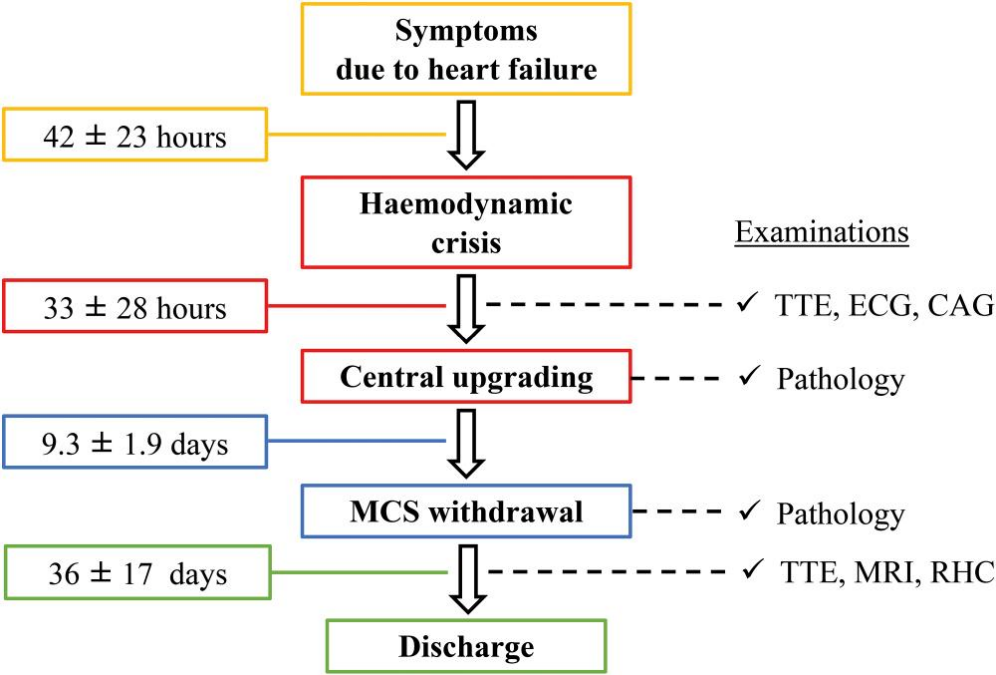
Timeline of treatment from symptom onset to hospital discharge. CAG, coronary angiography; ECG, electrocardiography; MRI, magnetic resonance imaging; RHC, right heart catheterization; TTE, transthoracic echocardiography.

## Patient information

The demographics, pre- and post-operative MCS details, haemodynamic data, and clinical outcomes of the patients are summarized in *Tables 1–4*.

**Table 1:** Perioperative characteristics of patients

Characteristic	Patient 1	Patient 2	Patient 3	Patient 4
Age (years)	30	38	43	65
Sex	Male	Female	Female	Male
BSA (m^2^)	2.31	1.63	1.65	1.74
Medical history	None	None	None	Hypertension, Hyperuricaemia
Presenting complaint	Severe dyspnoea	Persistent nausea and hypotension	Malaise	Hypotension
Peripheral MCS	VA-ECMO	VA-ECMO + Impella® CP	VA-ECMO + Impella® CP	VA-ECMO + IABP
LVEF before upgrading (%)	5	5	5	10
Peak CK-MB (U/L)	22	73	146	20
Peak troponin I (pg/mL)	119	19 300	17 700	15 400
Peak troponin T (ng/mL)	0.385	1.78	3.41	2.9
Peak creatinine (mg/dL)	2.04	2.09	2.49	4.2
Peak AST (U/L)	63	441	771	3790
Peak ALT (U/L)	124	75	410	3251
Central MCS	t-BiVAD	t-BiVAD	t-BiVAD	t-LVAD
Duration of central MCS (days)	12	8	8	9
LVEF after withdrawal (%)	53	60	60	60

ALT, alanine aminotransferase; AST, aspartate aminotransferase; BSA, body surface area; CK-MB, creatinine kinase–myocardial band; IABP, intra-aortic balloon pump; LVEF, left ventricular ejection fraction; MCS, mechanical circulatory support; t-BiVAD, temporary biventricular assist device; t-LVAD, temporary left ventricular assist device; VA-ECMO, venous–arterial extracorporeal membrane oxygenation.

**Table 2:** Haemodynamic data prior to upgrading of mechanical circulatory support

Patient	Peripheral MCS	Catecholamines (µg/kg/min)	HR (b.p.m.)	MBP (mmHg)	MPAP (mmHg)	CVP (mmHg)
1	VA-ECMO	Noradrenaline 0.2 Dobutamine 3	99	61	23	9
2	VA-ECMO + Impella® CP	Dopamine 10 Dobutamine 3	110	63	16	13
3	VA-ECMO + Impella® CP	Dopamine 5 Dobutamine 5	141	60	16	16
4	VA-ECMO + IABP	Vasopressin 2 (U/h) Noradrenaline 0.2 Dobutamine 2.5	150	60	18	18

CVP, central venous pressure; HR, heart rate; IABP, intra-aortic balloon pump; MBP, mean blood pressure; MCS, mechanical circulatory support; MPAP, mean pulmonary artery pressure; VA-ECMO, venous–arterial extracorporeal membrane oxygenation.

**Table 3:** Peripheral mechanical circulatory support data prior to upgrading

Patient	Peripheral MCS	BSA (m^2^)	BMI (kg/m^2^)	ECMO flow (L/min/m^2^)	Impella® flow (L/min)	ECMO outflow cannula (Fr)	ECMO inflow cannula (Fr)
1	VA-ECMO	2.3	33.5	3.5	—	20	24
2	VA-ECMO + Impella® CP	1.6	23.8	3.8	0.5	19	21
3	VA-ECMO + Impella® CP	1.7	30.9	2.4	3.0	16.5	21
4	VA-ECMO + IABP	1.7	27.8	3.5	—	13.5	21

BMI, body mass index; BSA, body surface area; ECMO, extracorporeal membrane oxygenation; IABP, intra-aortic balloon pump; MCS, mechanical circulatory support; VA-ECMO, venous–arterial extracorporeal membrane oxygenation.

**Table 4:** Clinical outcomes

Patient	RVAD withdrawal (days)	LVAD withdrawal (days)	Extubation (days)	MCS complications	Comorbidities	Hospital stay (days)
1	4	12	7	None	None	46
2	8	8	11	None	Haemorrhagic stroke	32
3	7	8	11	Limb neuropathy (VA-ECMO)	Acute kidney injury	32
4	—	9	10	None	Interstitial pneumonia Acute kidney injury Upper GI bleeding	69

RVAD, right ventricular assist device; LVAD, left ventricular assist device; MCS, mechanical circulatory support; VA-ECMO, venous–arterial extracorporeal membrane oxygenation; GI, gastrointestinal.

### Patient 1

A 30-year-old male patient with no remarkable medical history was hospitalized with severe dyspnoea after being infected with COVID-19. One month before presenting with symptoms of heart failure, he was admitted to a residential recovery facility with fever and a positive result for SARS-CoV-2 on reverse transcription polymerase chain reaction (RT–PCR) nasal swab testing. In the physical examination, the patient showed pitting oedema in the lower extremities and an irregular rhythm. A transthoracic echocardiograph (TTE) revealed a severely reduced left ventricular ejection fraction (LVEF) with a left ventricular outflow tract velocity time integral of 8.5 cm. Electrocardiographs showed tachycardia due to atrial fibrillation. However, no signs of pneumonia were observed on chest computed tomography. The following morning, he experienced severe hypotension and tachycardia after complaining of chest pain, and he underwent oral intubation and introduction of venous–arterial extracorporeal membrane oxygenation (VA-ECMO). Coronary angiographs (CAGs) showed no evidence of ischaemia. Despite initiating VA-ECMO with a 3.7 L/min flow, the haemodynamics were unstable, and renal and hepatic function progressively worsened, as indicated by elevated creatinine, aspartate aminotransferase, and alanine aminotransferase levels shown in *Table 1*. The end-organ dysfunction probably worsened because of insufficient blood flow, as the patient had a large body size. The patient was referred to our centre for optimal upgrading of MCS.

On admission, a TTE revealed severely reduced ventricular function and an aortic valve that was constantly closed. After discussions among the emergency heart team, upgrading to central MCS was planned to achieve both adequate blood flow and ventricular unloading, as the diameter for peripheral vascular access was restricted. We followed our institutional protocol^2^ for surgery for central cannulation and checked right ventricular (RV) function by connecting the left ventricular (LV) drainage cannula to the cardiopulmonary circuit. We thus observed that the LV preload was deficient due to severe RV failure. These circumstances caused unacceptable suction in the LV vent; we added an outflow graft to the pulmonary artery to prevent LV thrombus formation, establishing a central biventricular support (BiVAD) using an extracorporeal centrifugal cardiopulmonary assist system, BIOFLOAT-NCVC®, and BIOCUBE® (NIPRO, Osaka, Japan). Four days after implantation, weaning from RV support was successful and the system was converted to an extracorporeal LV assist device (LVAD). Left ventricular function gradually recovered with LV unloading. Surgery, including weaning from the LVAD, pulmonary vein isolation, and left atrial appendage closure, was performed 12 days after the initial operation. After removing the LVAD, all vasopressor infusions were titrated and tapered off.

One month after withdrawal, a TTE demonstrated an LVEF of 53%, and a right heart catheterization (RHC) study revealed normal haemodynamics with a cardiac index of 2.6 L/min/m^2^. Cardiac magnetic resonance images revealed normal biventricular contraction [LVEF, 59.6%; RV ejection fraction (RVEF), 51.8%]; myocardial fibrosis was apparent only at the cannulation site on the LV apex on late gadolinium enhancement imaging. He was discharged independently and he returned to daily life, remaining in good condition at the 2-year follow-up.

### Patient 2

A 38-year-old female patient, previously healthy, was taken to hospital by ambulance with persistent nausea and hypotension after SARS-CoV-2 was detected on RT–PCR testing of a nasal swab on the same day. During the physical examination, she exhibited signs of cardiogenic shock, including cold extremities and weak peripheral pulses. ST-segment elevation in the inferior leads, serum troponin-T levels elevated to 2.4 ng/mL, and diffuse LV hypokinesis on TTE suggested acute myocarditis. The patient was referred to another hospital, where her haemodynamics worsened, resulting in pulseless electrical activity on electrocardiography. An emergency initiation of VA-ECMO and insertion of an intra-aortic balloon pump (IABP) were performed after 30 min of cardiopulmonary resuscitation. The IABP was upgraded to the Impella® CP (Abiomed, Danvers, MA, USA) at another medical centre. However, this device was unable to provide adequate haemodynamic stability because of consistent suction alarms, resulting in a progression of end-organ dysfunction. Despite the initiation of empirical treatment with methylprednisolone 1 g (3 days) and intravenous immunoglobulin 1 g/kg/day (2 days) for myocarditis, her LV function and haemodynamics consistently worsened. Finally, the patient was referred to our centre for further upgrading of MCS.

On admission, a TTE revealed an LVEF of 5% with a small LV cavity (LV end-diastolic diameter, 34 mm) and severely reduced RV contraction. A computed tomography scan and laboratory data (*Table 1*) demonstrated severe myocardial injury and multiple organ dysfunction that was deteriorating. As for Patient 1, we upgraded MCS to extracorporeal BiVAD, achieving an aortic return of 4.5 L/min (3.0 L/min/m^2^). Dexamethasone 3.3 g/day and molnupiravir were used to treat the COVID-19 infection. Biventricular unloading was remarkably effective, with both LV and RV functions improving steadily. Thus, the biventricular support was removed on post-operative Day 8 and all inotropes were smoothly tapered.

Due to acute renal failure, the patient required continuous haemodiafiltration during BiVAD support. Haemodiafiltration was successfully withdrawn after BiVAD removal. Follow-up TTE and RHC studies were conducted 3 weeks after withdrawal, and no evidence of LV dysfunction was observed. The LVEF was 60% and cardiac index 2.5 L/min/m^2^ without inotropes. Cardiac magnetic resonance images revealed normal biventricular contraction (LVEF, 53.5%; RVEF, 55.1%), and myocardial fibrosis was apparent only at the cannulation site on the LV apex. She was discharged independently and she returned to daily life, remaining in good condition at the 2-year follow-up.

### Patient 3

A 43-year-old female, previously healthy, complained of malaise. She was referred to a medical centre, presenting with cardiogenic shock, when COVID-19 infection was confirmed by a positive result for SARS-CoV-2 on RT–PCR testing of nasal swabs. During the physical examination, she had elevated jugular venous pressure, and crackles were observed. Severely reduced contraction in both ventricles on TTE, ST-segment elevation in multiple leads on electrocardiography, and intact coronary arteries on CAG suggested acute myocarditis. The Impella® CP (Abiomed) failed to stabilize her haemodynamics: pulmonary artery wedge pressure (20 mmHg) and central venous pressure (20 mmHg) were both elevated. Venous–arterial extracorporeal membrane oxygenation was therefore introduced, and the patient was referred to our centre for upgrading of MCS.

Due to insufficient total blood flow, a restricted diameter of the peripheral arteries, and vascular access complications, upgrading to a central system was decided, and extracorporeal BiVAD was introduced, as for Patients 1 and 2. Sotrovimab and molnupiravir were administered to treat the COVID-19 infection, and intravenous immunoglobulin 1 g/kg/day (3 days) and methylprednisolone 1 g (3 days) were also administered. The patient required considerable intravenous fluid administration for ∼4 days, probably because of a cytokine storm associated with the COVID-19 infection.

Continuous haemodiafiltration was initiated on post-operative Day 3 to treat progressive renal failure. Conversely, biventricular function recovered smoothly after unloading, and we removed the RVAD and LVAD on post-operative Days 7 and 8, respectively (*Table 4*). Transthoracic echocardiography and RHC examinations were conducted 2 weeks after device withdrawal and the findings showed that biventricular function was normal (*Table 1*). She was discharged independently and she returned to daily life, remaining in good condition at the 1.5-year follow-up.

### Patient 4

A 65-year-old male with a history of hypertension and hyperuricaemia was diagnosed with COVID-19 and admitted to an emergency room with hypotension 5 days later. During the physical examination, he presented with severe dyspnoea, bilateral rales, and pitting oedema in the lower extremities. A TTE revealed a generally reduced ejection fraction and slight pericardial effusion. No significant stenosis in the coronary arteries was observed on CAG. His haemodynamics progressively deteriorated with atrial fibrillation, even after an IABP and VA-ECMO were inserted. The patient was referred to our centre 2 days after the initial admission.

A transoesophageal echocardiography showed a severely reduced ejection fraction of <10%, and we decided to upgrade MCS to the central system to control progressive multiple organ dysfunction and pulmonary congestion under limited percutaneous vascular access. Unlike the previous cases of patients, sufficient flow of >5.0 L/min (2.8 L/min/m^2^) could be maintained with extracorporeal LVAD without RV support. Remdesivir was administered to treat the COVID-19 infection. After empirical treatment with intravenous immunoglobulin 1 g/kg/day (5 days) and methylprednisolone 1 g (3 days), LV function gradually improved. Nine days after the initial operation, we performed LVAD withdrawal, a maze procedure, and left atrial appendage closure. After LVAD removal, dobutamine was successfully tapered over a week.

Echocardiography was performed 3 weeks after LVAD withdrawal and scans showed normal LV function (LVEF, 60%). Although the patient was extubated the following day, re-intubation was required within a week to treat a deterioration in respiratory function. A computed tomography scan revealed acute exacerbation of interstitial pneumonia, which might have been caused by COVID-19, and the patient underwent a tracheotomy 3 weeks after LVAD withdrawal. He was transferred to a rehabilitation facility after a 2-month hospitalization and, although he developed bacterial pneumonia, he was discharged after 3 months of rehabilitation with no issues in cardiac function.

### Histopathological findings

**Figure 1:**
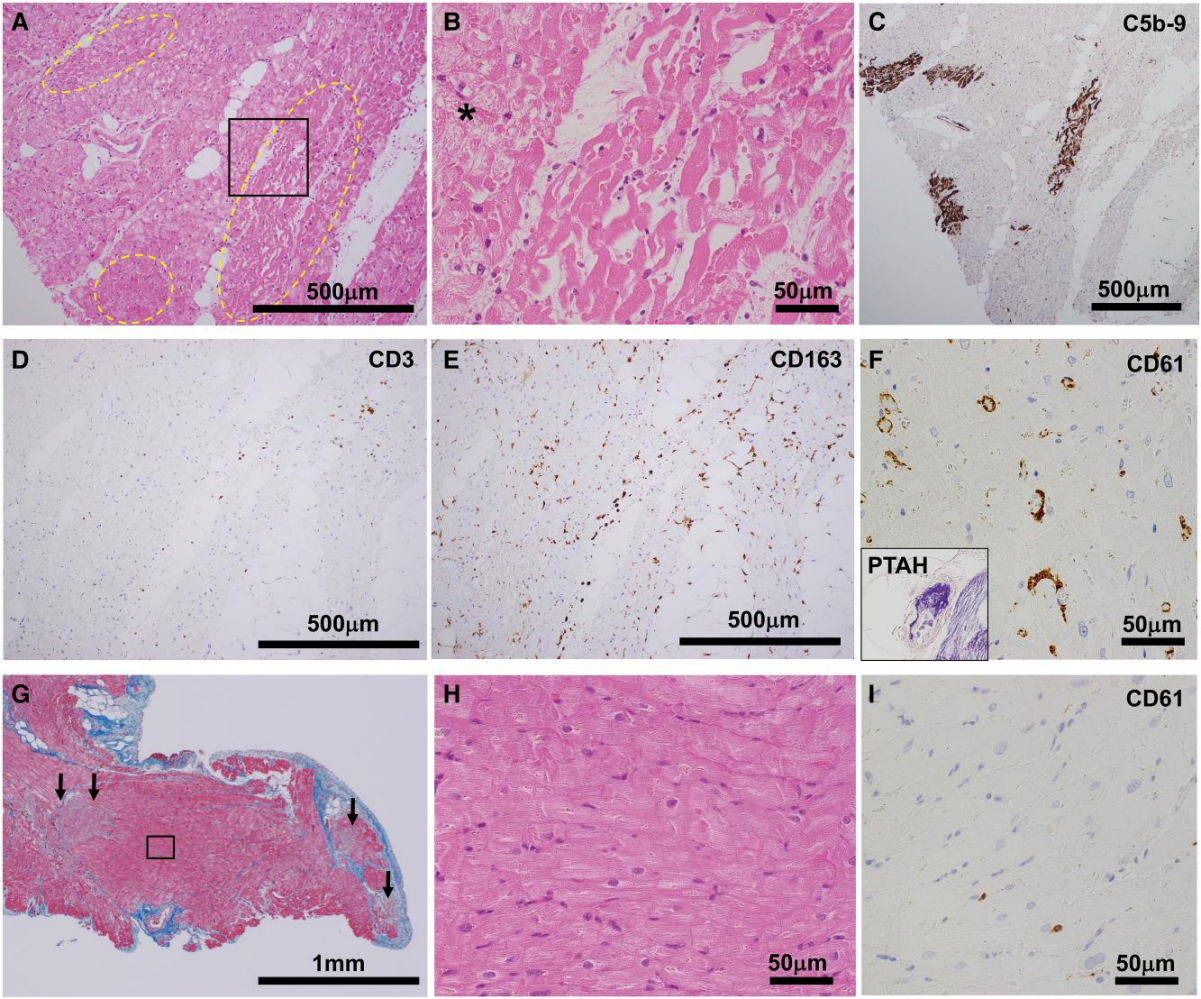
Representative histopathological findings (Patient 4) of the left ventricular apex obtained during the initial and withdrawal surgeries. (*A–F*) Specimens obtained during initial surgery. A low-power view of the myocardium with patchy areas of coagulative necrosis (dashed area in *A*). Haematoxylin and eosin staining (*A*). A high-power view of the border zone of the necrotic area (*B*, rectangle in *A*). Fine cytoplasmic vacuolization is observed in viable myocytes (asterisk). Immunostaining for C5b-9 showed necrotic areas (*C*). Sparse T lymphocytes (*D*, immunostaining for CD3) and increased interstitial macrophages (*E*, immunostaining for CD163) are shown. Immunostaining for CD61 showing increased platelet aggregation in the microvessels (*F*). The inset shows the fibrin microthrombi in a small vessel; phosphotungstic acid–haematoxylin (PTAH) stain. (*G–I*) Specimens obtained during surgery to withdraw the device. A low-power view of the myocardium with small necrotic areas in the sub-acute, reparative stage (arrows; *G*, Masson’s trichrome stain). Compared with the initial surgical specimens, the remaining myocytes showed no apparent fine cytoplasmic vacuolization (*H*) and contained fewer microthrombi in the small vessels (*I*, immunostaining against CD61).

Histopathological specimens were surgically obtained from the apex of the LV, both in the initial surgeries for implanting the central MCS and in the withdrawal surgeries. Initial surgery specimens revealed small wavy areas of early ischaemic coagulative necrosis (*Figure 1A–C*) in two patients, with viable myocytes showing diffuse cytoplasmic vacuolization (*Figure 1B*, asterisk). No features of conventional fulminant myocarditis, such as lymphocytic, eosinophilic, or giant cell myocarditis, were observed. Immunohistochemical examinations confirmed sparse CD3^+^ T cells (*Figure 1D*) with diffusely increased interstitial CD68+ and CD163^+^ macrophages (*Figure 1E*) in all patients. Three patients exhibited intravascular CD61+ platelet aggregation, and one exhibited fibrin microthrombi as well as platelet aggregation (*Figure 1F*, inset). Small vessels showed plump endothelial cells, and megakaryocytes were rarely observed within the capillary lumen. Frozen heart tissue specimens obtained from all the patients underwent a multi-virus real-time PCR assay at the National Institute of Infectious Diseases; the analysis showed no viral genomes, including those of SARS-CoV-2.^3^ In the withdrawal surgery specimens, except for small necrotic areas of the sub-acute reparative stage (*Figure 1G*, arrows), the remaining myocardium showed no obvious cytoplasmic vacuolization (*Figure 1H*) and reduced microthrombi (*Figure 1I*).

## Discussion

In these four patients with COVID-19-associated refractory cardiogenic shock, (i) rapid haemodynamic deterioration occurred after initial signs of acute heart failure and required prompt peripheral MCS, and (ii) within 2 weeks of inducing central MCS, ventricular function significantly improved from near-complete cardiac standstill to normal ventricular contraction.

Most patients with COVID-19 develop cardiovascular complications such as acute cardiac injury with elevated troponin levels, heart failure, pericardial effusion, and acute myocarditis.^4^ Cases of refractory cardiogenic shock requiring MCS have been reported.^5–10^ Early recovery can be achieved using percutaneous MCS, such as VA-ECMO, IABP, or Impella®, in patients with a relatively preserved RV function, no progressive pulmonary congestion, and comfortable peripheral vascular access.^6,8^ However, some patients with severely reduced LVEF and prolonged haemodynamic instability die as a result of VA-ECMO complications and multiple organ failure,^7,10^ whereas others require an implantation of durable LVAD^9^ or heart transplantation^5^ to survive. In our patients, because of the restricted diameter of the peripheral vessels, severe RV failure, and pulmonary hypertension, we upgraded MCS to the central system. The prompt and reliable ventricular unloading achieved through central systems led to a steady improvement in ventricular function, even with initial severe biventricular dysfunction. In our patients, due to anatomical limitations, inadequate LV preload, and unavailability of peripheral RV support devices in Japan, the upgrade from Impella® CP to 5.5 was not feasible. The survival of a patient with the simultaneous use of Impella® 5.0 and Impella® RP has been reported.^11^ Central MCS can be a more invasive approach, as evidenced by one of our patients requiring re-exploration to remove haematomas causing low flow. Although this additional procedure did not negatively impact the final outcome, it highlights the importance of considering less-invasive peripheral approaches whenever feasible.

Unlike conventional fulminant myocarditis, the myocardial specimens of our patients showed macrophage-predominant interstitial infiltration with sparse T lymphocytes, similar to previously reported findings in an autopsied heart with COVID-19.^12^ Furthermore, in all patients, we found features of microthrombosis with endothelial activation, which is described as an important mechanism of myocardial injury in COVID-19.^7,13^ Scattered coagulative necrotic areas were not associated with lymphocyte accumulation but with ischaemic origin at the microvascular level. Intriguingly, microthrombi and fine cytoplasmic vacuolization in viable cardiomyocytes, which were observed in the initial surgery, almost disappeared upon withdrawal, indicating that despite decreased contractility due to a temporarily impaired microvascular integrity, myocardial viability may remain unaffected. Other mechanisms, such as elevated cytokine levels, could also influence temporary deterioration in myocardial contractility.

When comparing our results with the ECLS-SHOCK trial,^14^ which investigated the use of peripheral extracorporeal life support devices in patients with infarct-related cardiogenic shock, our patients exhibited two distinct differences. Firstly, the aetiological backgrounds differ. In our four patients, cardiac muscle viability was preserved despite acute severe deterioration. Therefore, surviving the cardiac stunning period without severe end-organ dysfunction is crucial for a better prognosis. Our aggressive strategy to maintain temporary haemodynamics was tailored to the specific aetiology and it was successful. In ischaemic patients, however, cardiac viability can be irreversibly compromised, meaning that achieving temporary haemodynamic stability may not translate into better outcomes. Secondly, we transitioned from peripheral to central MCS due to anatomical concerns. Insufficient flow from the peripheral MCS can exacerbate end-organ dysfunction and hinder myocardial recovery. In contrast, central MCS provided sufficient flow and effective ventricular unloading, thereby preventing the occurrence of severe complications like peripheral bleeding and limb ischaemia.

In conclusion, when treating COVID-19-associated refractory cardiogenic shock, prompt MCS to achieve biventricular unloading may enable the management of acute haemodynamic deterioration and steady recovery of biventricular function without progression to persistent end-organ dysfunction.

## Lead author biography


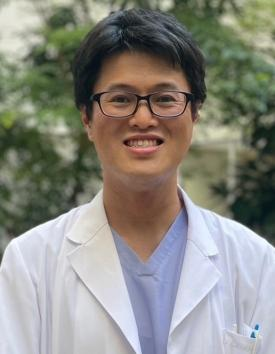


Kohei Tonai, a cardiac surgeon at the National Cerebral and Cardiovascular Center (NCVC) in Osaka, Japan, specializes in advanced heart failure. He graduated from Osaka University Faculty of Medicine in 2016 and received cardiac surgery training at Osaka University Hospital, Sakakibara Heart Institute of Okayama, and NCVC. His research interests encompass extracorporeal membrane oxygenation, ventricular assist devices, heart transplantation, and cardiac regeneration therapies.

## Data Availability

The data pertaining to this case series can be made available upon request.
